# Supplementary material for: Human umbilical cord mesenchymal stem cells decellular matrix alleviates chondrocyte senescence by inhibiting the STING-NF-κB pathway
Source: PLoS One. 2025 Jun 26;20(6):e0325704. doi: 10.1371/journal.pone.0325704 (PMC12200692; doi:10.1371/journal.pone.0325704)
Supplement: S1 Table — (DOCX) [file pone.0325704.s001.docx]

| **Table S1.** Primer sequences used in this study | | |
| --- | --- | --- |
| **Gene name** | **F/R** | **Sequences 5'-3'** |
| IL-6 | F | AAATCTGCTCTGGTCTTC |
|  | R | AGGGTTTCAGTATTGCTC |
| COX-2 | F | GTTCCAACCCATGTCAAAACCGT |
|  | R | GGCCCTGGTGTAGTAGGAGAGGT |
| iNOS | F | CAGGCTGGAAGCCGTAACAA |
|  | R | CGATGCACAACTGGGTGAAC |
| MMP3 | F | GTCCAGAAGATCGATGCAGC |
|  | R | TCCAACTGTGAAGATCCGCT |
| MMP13 | F | GCCACCTTCTTCTTGTTGAGTTG |
|  | R | GACTTCTTCAGGATTCCCGCA |
| ADAMTS5 | F | ACAAGAGTCTGGAGGTGAGCAAG |
|  | R | ACATATGGTCCCAACGTCTGC |
| COL2a1 | F | GACTGTGCCTCGGAAGAA |
|  | R | CTGGACGTTAGCGGTGTT |
| AGGRECAN | F | AGTGACCCATCTGCTTACCCTG |
|  | R | CTGCATCTATGTCGGAGGTAGTG |
| β-actin | F | TGCTATGTTGCCCTAGACTTCG |
|  | R | GTTGGCATAGAGGTCTTTACGG |
|  |  |  |
